# Monitoring COVID-19 and Influenza: The Added Value of a Severe Acute Respiratory Infection Surveillance System in Portugal

**DOI:** 10.1155/2023/6590011

**Published:** 2023-02-16

**Authors:** Ana Rita Torres, Verónica Gómez, Irina Kislaya, Ana Paula Rodrigues, Margarida Fernandes Tavares, Ana Catarina Pereira, Débora Pereira, Rita Côrte-Real, Carlos Humberto Flores, Nuno Verdasca, Raquel Guiomar, Ausenda Machado

**Affiliations:** ^1^Department of Epidemiology, National Health Institute Doutor Ricardo Jorge, Portugal; ^2^Public Health Research Center, NOVA National School of Public Health, Lisbon, Portugal; ^3^Comprehensive Health Research Center, Lisbon, Portugal; ^4^Centro Hospitalar Universitário de São João, Porto, Portugal; ^5^Centro Hospitalar Universitário Lisboa Central, Lisbon, Portugal; ^6^Department of Infectious Diseases, National Health Institute Doutor Ricardo Jorge, Lisbon, Portugal

## Abstract

**Background:**

Severe acute respiratory infections (SARI) surveillance is recommended to assess the severity of respiratory infections disease. In 2021, the National Institute of Health Doutor Ricardo Jorge, in collaboration with two general hospitals, implemented a SARI sentinel surveillance system based on electronic health registries. We describe its application in the 2021/2022 season and compare the evolution of SARI cases with the COVID-19 and influenza activity in two regions of Portugal.

**Methods:**

The main outcome of interest was the weekly incidence of patients hospitalized due to SARI, reported within the surveillance system. SARI cases were defined as patients containing ICD-10 codes for influenza-like illness, cardiovascular diagnosis, respiratory diagnosis, and respiratory infection in their primary admission diagnosis. Independent variables included weekly COVID-19 and influenza incidence in the North and Lisbon and Tagus Valley regions. Pearson and cross-correlations between SARI cases, COVID-19 incidence and influenza incidence were estimated.

**Results:**

A high correlation between SARI cases or hospitalizations due to respiratory infection and COVID-19 incidence was obtained (*ρ* = 0.78 and *ρ* = 0.82, respectively). SARI cases detected the COVID-19 epidemic peak a week earlier. A weak correlation was observed between SARI and influenza cases (*ρ* = −0.20). However, if restricted to hospitalizations due to cardiovascular diagnosis, a moderate correlation was observed (*ρ* = 0.37). Moreover, hospitalizations due to cardiovascular diagnosis detected the increase of influenza epidemic activity a week earlier.

**Conclusion:**

In the 2021/2022 season, the Portuguese SARI sentinel surveillance system pilot was able to early detect the COVID-19 epidemic peak and the increase of influenza activity. Although cardiovascular manifestations associated with influenza infection are known, more seasons of surveillance are needed, to confirm the potential use of cardiovascular hospitalizations as an indicator of influenza activity.

## 1. Background

In response to SARS-CoV-2 emergence, substantial changes occurred in respiratory infection surveillance activities at inpatient and outpatient levels, moving the focus from disease specific surveillance of influenza and other respiratory viruses into SARS-CoV-2 [1]. With unprecedented nonpharmacological measures in place to control the COVID-19 pandemic, a decrease in other respiratory virus incidence was observed worldwide, with low or almost inexistent circulation of influenza during 2020/2021 compared with previous seasons. However, in the 2021/2022 season, the circulation of influenza resumed and the European region, including Portugal, experienced a late epidemic activity compared to most previous seasons [2, 3].

Besides anomalous patterns of influenza and other respiratory virus circulation, several countries also reported an increase in the severity of disease, highlighting the need for robust, sustainable, comprehensive, and near real-time surveillance of severe respiratory infection diseases to guide public health response [4, 5]. Currently, following the World Health Organization (WHO) and the European Centre for Disease Prevention and Control (ECDC) recommendations, several countries are transitioning from COVID-19 emergency to routine surveillance and integrated severe acute respiratory infections (SARI) surveillance for hospital inpatients [6]. Collecting timely data on respiratory infections is essential to understand their impact on population and healthcare services, and to inform mitigation measures. In particular, SARI surveillance systems are needed to compare the severity of different seasons, to monitor groups at high risk of severe disease and complications from respiratory infections, and to track the viruses, which specifically cause severe disease [7].

Syndromic register-based surveillance using hospital admissions' information can be an efficient approach to detect changes in disease patterns and create early alerts on its potential impact, without additional data collection burden [8]. In Germany, results from a syndromic SARI surveillance system provided (i) timely and reliable information on the influenza virus circulation, between 2011/2012 and 2015/2016 seasons, (ii) enabled valid estimation of COVID-19 hospitalization incidence in 2020/2021, and (iii) revealed underreporting of hospitalizations during the peaks of three COVID-19 waves [9, 10]. Syndromic intensive care unit (ICU) SARI surveillance in the Netherlands, provided insight into the severity of influenza epidemics, between 2007 and 2016 [11]. In the early stages of the pandemic, in Portugal, a syndromic surveillance pilot for respiratory infections, based on electronic health registry of hospital admissions, found that hospitalizations due to viral pneumonia detected variations in the frequency of the COVID-19 incidence for about a week earlier, suggesting that this indicator can early detect COVID-19 outbreaks [12].

At national level, in 2021, within the European Union SARI surveillance network (E-SARI-Net), the National Institute of Health Doutor Ricardo Jorge, in collaboration with two general hospitals, evaluated the feasibility of implementing a near real-time, semiautomated, SARI sentinel surveillance system based on electronic health registries [6]. This passive, surveillance system is based on impatient data, coded at admission according to the International Statistical Classification of Diseases and Related Health Problems 10^th^ Revision (ICD-10), corresponding to diagnoses related to the clinical syndrome SARI [13]. The clinical syndrome SARI includes signs and symptoms related to influenza and other respiratory infections, acute cardiovascular events commonly associated with respiratory infections, and exacerbation of chronic respiratory disease [9, 14–19].

In this work, we describe the establishment of the Portuguese SARI sentinel surveillance system and its application in the 2021/2022 season (week 40 2021 to week 20 2022). Additionally, we compare the evolution of SARI cases with the COVID-19 and influenza activity in two regions of Portugal.

## 2. Methods

### 2.1. Setting and Study Design

This is a retrospective surveillance study implemented in two Portuguese university general hospitals, Centro Hospitalar Universitário de São João and Centro Hospitalar Universitário Lisboa Central, located in the main cities (Oporto and Lisbon, respectively) of the two most populated regional health administrations (North and Lisbon and Tagus Valley regions, respectively) of the country. These sentinel sites were selected among the I-MOVE (Influenza–Monitoring Vaccine Effectiveness in Europe) hospital network and the Portuguese Laboratory Network for the Diagnosis of Influenza Infection [20–23]. Detailed information on the participating hospitals and respective wards is provided in Table 1.

### 2.2. SARI Case Identification

**Figure 1:**
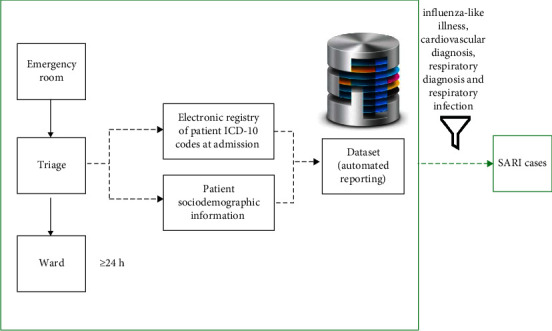
Severe acute respiratory infections surveillance system flowchart. The patient circuit is identified using solid arrows; electronic information flow is identified using dashed arrows. SARI: severe acute respiratory infection.

SARI cases were identified among those who have been admitted for at least 24h, in one of the participating hospitals. In the emergency room (ER) a diagnosis at admission, coded according to the ICD-10, is registered by the ER team for every patient [13]. Anonymised case-based data regarding inpatients admitted to all wards were made available on a weekly basis by the sentinel hospitals and included patients' sex, age, date of admission, and primary admission diagnosis. All reporting procedures for each hospital are automated, routinely programmed, and in accordance with legal and ethical requirements. SARI cases were defined as patients containing any ICD-10 codes for influenza-like illness, cardiovascular diagnosis, respiratory diagnosis, and respiratory infection in their primary admission diagnosis. These ICD-10 codes were selected after a literature research and based on the Integrated Monitoring of Vaccines in Europe project (I-MOVE+) (see Table 2 for ICD-10 codes) [9, 14–19]. We categorized the most common signs and symptoms related to influenza infection into the influenza-like illness group, while acute cardiovascular events commonly associated with respiratory infections were categorized into cardiovascular diagnosis, and the most common respiratory infections were categorized into the respiratory infections group. All remaining respiratory signs and symptoms and exacerbation of chronic respiratory disease (e.g., asthma) were categorized into the respiratory diagnosis group. The generic surveillance system flow is presented in Figure 1.

The SARI sentinel surveillance system protocol was approved by the Ethical Committees of the National Institute of Health Doutor Ricardo Jorge, Centro Hospitalar Universitário de São João, and Centro Hospitalar Universitário Lisboa Central. Given that data used within this study were pseudoanonymised and collected in the scope of epidemiological surveillance of respiratory viruses with epidemic or pandemic potential, such as influenza and SARS-CoV-2, the need for the participants' informed consent was waived by the Ethical Committees [24]. Patient data were pseudoanonymised by the information technology personnel at each sentinel site, by removing identifiable features: name, date of birth, address, and telephone numbers. The 9-digit Portuguese National Health Service IDs were replaced with a unique and anonymous code. The key to map Portuguese National Health Service IDs to anonymous IDs was secured at each sentinel site.

### 2.3. Population under Surveillance

The population under surveillance consists in all individuals living in the catchment area of each sentinel site, who would usually seek healthcare at the site when they get sick. We reviewed the hospital discharge registry database (BIMH–Identity Card for Hospital Morbidity) that covers all admission in public hospitals in Portugal mainland, in order to prepare a hot spot map based on SARI cases according to the place of residency. This map corresponded to at least 80% of SARI cases hospitalized at each sentinel site in the last three years [25]. For each selected municipality within the spot map, we computed the proportion of SARI admitted by participating hospital among all SARI admissions registered in the municipality. Finally, to estimate individual contribution of each selected municipality to population under surveillance, we applied previously estimated proportions to most recent resident population figures for municipalities [26]. Please see Table 3 for detailed information on the population under surveillance for each sentinel site.

### 2.4. Variable Definitions

The primary outcome of interest was the weekly number of SARI cases, reported within the surveillance system. Secondary outcomes of interest included the age group and diagnosis at admission for SARI cases. Independent variables of interest included the weekly COVID-19 and influenza incidence in the North and Lisbon and Tagus Valley regions. Surveillance data on weekly COVID-19 laboratory-confirmed cases were made available by the Directorate General of Health (DGS) in Portugal [27]. The number of weekly positive samples for influenza was made available by the Portuguese Laboratory Network for the Diagnosis of Influenza Infection [21–23]. If our surveillance system was useful to monitor COVID-19 and influenza activity, then we expected to see a significant association between SARI cases and COVID-19 and influenza incidence.

### 2.5. Study Period

All data were updated on 22 July 2022, and data between week 40 2021 (2021/40) and week 20 2022 (2022/20) (comprising the period from 28 September 2021 to 22 May 2022) was used as observation period, for analyzing outcomes of the surveillance system and for comparing SARI cases with the COVID-19 and influenza activity.

### 2.6. Statistical Analyses

Descriptive statistics (count and percentages) were used to characterize distribution of SARI cases reported by surveillance system. A time-series analysis was performed using the weekly number of SARI cases and two indicators: (1) the weekly number of samples positive for SARS-CoV-2 and (2) the weekly number of samples positive for influenza. Pearson correlation (0 ≤ *ρ* < 0.3-weak correlation; 0.3 ≤ *ρ* < 0.7-moderate correlation; and 0.7 ≤ *ρ*-strong correlation) and cross-correlations between SARI cases and each indicator were estimated [28].

The cross-correlation study allows the identification of the lag (delay) between two time-series. If a higher cross-correlation value is found on lag 0, the values of the first series (SARI cases) are correlated with the values of the second series (COVID-19 cases or influenza positive samples) without delay. If the higher cross-correlation value is found to have a negative lag, the values of the first series are correlated with the values of the second series with a delay of lag weeks, and if the higher cross-correlation value is found to have a positive lag, the values of the first series are correlated with the values of the second series, and the second series precedes the first in lag weeks [29].

All analyses were performed using *R* 4.1.2 statistical software [30].

## 3. Results

**Figure 2:**
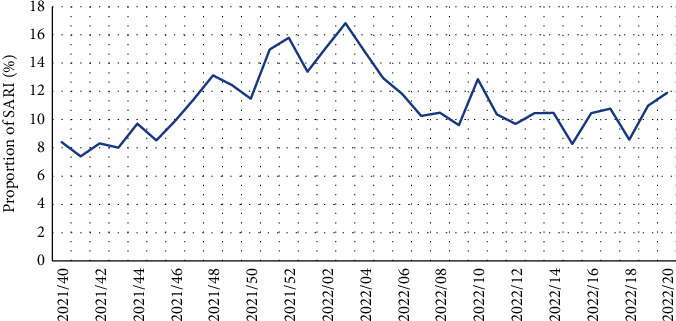
Severe acute respiratory infection weekly proportion to all hospital admissions, North and Lisbon and Tagus Valley regions, 28 September 2021–22 May 2022 (number of SARI = 3,563; number of total hospital admissions = 32,011). SARI: severe acute respiratory infection.

From a total of 32,011 hospital admissions that were notified by the participating hospitals, between week 40 2021 and week 20 2022, 3,563 encompassed SARI-related diagnoses corresponding to 11.1% of all hospital admissions and to a cumulative SARI incidence rate of 394.1 per 100,000 population. However, the proportion of SARI cases varied during the study period, ranging from 7.4% on week 41 2021 to 16.8% on week 03 2022 (see Figure2).

**Figure 3:**
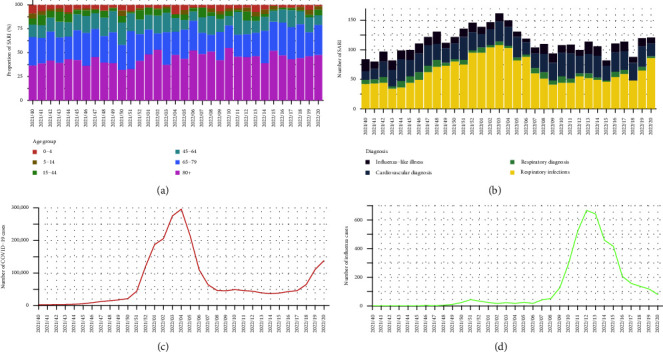
(a) Distribution of weekly severe acute respiratory infections by age group, North and Lisbon and Tagus Valley regions, 28 September 2021–22 May 2022 (*n* = 3,563), (b) weekly counts of severe acute respiratory infections stratified by diagnosis, North and Lisbon and Tagus Valley regions, 28 September 2021–22 May 2022 (*n* = 3,563), (c) weekly counts of COVID-19 cases, North and Lisbon and Tagus Valley regions, 28 September 2021–22 May 2022 (*n* = 2,367,770), and (d) weekly counts of influenza cases, North and Lisbon and Tagus Valley regions, 28 September 2021–22 May 2022 (*n* = 4,174). SARI: severe acute respiratory infection; COVID-19: coronavirus disease.

The general characteristics of the SARI cases are summarised in Table 4. The age group (4.5% ≤ 0–4 years; 2.5% 5–14 years; 5.2% 15–44 years; 15.9% 45–64 years; 28.4% 65–79 years; and 43.7% ≥ 80 years) and diagnosis distributions (12.8% influenza-like illness; 27.7% cardiovascular diagnosis; 6.4% respiratory diagnosis; and 58.4% respiratory infection) remained similar over the full observation period, but a relative increase in SARI in the age group over 80 years old was registered in weeks coincident with a high number of hospitalizations due to respiratory infections or cardiovascular diagnosis (Figure 3). Additionally, the pattern in admissions due to infections appears to follow the pattern of COVID-19 cases, whereas the increase in cardiovascular diagnosis (in weeks 10 and 11, 2022) appears to be time coincident with an increase in influenza cases (Figure 3).

Graphical patterns between SARI, COVID-19, and influenza cases was confirmed by cross-correlation analysis (Table 5). A high correlation between SARI cases or hospitalizations due to respiratory infection and COVID-19 incidence was obtained (*ρ* = 0.78 and *ρ* = 0.82, respectively). Moreover, weekly SARI cases detected COVID-19 epidemic peak for about a week earlier (highest cross-correlation value for lag = −1). A weak correlation was observed between SARI and influenza cases (*ρ* = −0.20). However, if restricted to hospitalizations due to cardiovascular diagnosis, a moderate correlation was observed (*ρ* = 0.37). Moreover, hospitalizations due to cardiovascular diagnosis detected the increase of influenza epidemic activity for about a week earlier (highest cross-correlation value for lag = −1). This early warning was not observed between hospitalizations due to cardiovascular diagnosis and COVID-19 cases.

## 4. Discussion

Our analyses demonstrated the potential of a register-based SARI hospital sentinel surveillance system. The temporal pattern of SARI cases, in particular, patients with respiratory infection diagnosis, corresponded well to the course of the COVID-19 incidence in the regions of North and Lisbon and Tagus Valley. In addition, the SARI surveillance system was able to early detect the COVID-19 epidemic peak, in January 2022. These results are also consistent with COVID-19 hospitalization data for the North and Lisbon and Tagus Valley regions, which peaked in weeks 3‒5 2022 with 1,606 beds per week occupied in hospital wards on average [31]. Monitoring hospitalizations due to viral pneumonia had already proven to be an asset regarding the early detection of COVID-19 outbreaks in Portugal during the early stages of the pandemic, which is in line with results from this work [12].

We note that the modest increase observed in SARI cases comparing to the steep increase in COVID-19 cases, from week 50 2021 onwards, is consistent with the increased transmissibility and decreased pathogenicity of the Omicron variant [32]. In late December, Omicron swiftly replaced the then-dominant Delta variant in Portugal, resulting in a skyrocketing number of COVID-19 cases [33]. However, this increase did not result in a proportional hospital burden because, although Omicron has a 3.31-fold higher transmissibility than Delta, patients with Omicron infection are significantly less likely to be admitted to hospitals, require oxygen, or being admitted to ICUs [34, 35].

Hospitalizations due to cardiovascular diagnosis were able to early detect influenza activity in the North and Tagus Valley regions, suggesting this indicator might be used to monitor influenza activity. However, this early warning was not observed between hospitalizations due to cardiovascular diagnosis and COVID-19 cases. A review of the literature, also found peak influenza season to be associated with increased cardiovascular hospitalizations, contrasting with a decrease of approximately  50% in cardiovascular hospitalizations during the COVID-19 pandemic [36]. One possible explanation might be related to deferred care during times of high COVID-19 incidence, as several studies showed a significant decline in the number of patients seeking emergency care for cardiac events during the pandemic, citing hesitancy or fear as the main cause [37, 38]. In addition, the lack of exposure of the population to the influenza virus during the COVID-19 pandemic and the vaccination mismatch, might have resulted in increased severe forms of disease when infected with influenza, including cardiovascular complications during the 2021/2022 season [39–41]. Therefore, while the association between influenza and cardiovascular manifestations is known, more seasons of SARI surveillance are needed, to confirm the strength of the association between hospitalizations due to cardiovascular diagnosis and influenza activity [42].

Even though, the representativeness of data is a concern as paediatrics wards are only included in one of the sentinel sites, the distribution of SARI cases stratified by age, and the relative increase in the age group above 80 years old in periods with a high incidence of COVID-19 and influenza, was expected as older individuals have an increased risk of hospitalization during epidemic periods of respiratory infections [43, 44].

The findings of this study should be interpreted by taking the following limitations into account. First, the selected data are from two sentinel hospitals from 2 out of 5 health administration regions in the country, meaning that they are not representative of severe acute hospitalizations in Portugal. Second, using only primary diagnoses could capture mainly the population without major comorbidities, in particular, in hospitalizations due to influenza-like illness. SARI cases with underlying chronic disease (e.g., cardiovascular diseases) may have their comorbidity coded in the primary diagnosis and ICD-10 codes corresponding to symptoms of influenza-like illness in their secondary diagnosis, as the exacerbation of the chronic disease could play a major role in their hospitalization [42]. This is in line with the increase in hospitalizations due to cardiovascular diagnosis during the epidemic activity of Influenza in Portugal (between March and May 2022). Finally, the syndromic SARI case definition is based on the clinical documentation for the hospitalization at admission, which is not designed for surveillance. Its heavy reliance on clinical presentation, especially, when laboratory diagnosis is frequently not yet available, can create potential uncertainty in data interpretation. Shortness of breath, for example, may be a symptom resulting either from cardiovascular disease or from an infection by a respiratory pathogen [45].

The SARI surveillance system described in this study has several strengths that are worth highlighting. The sentinel sites included in this surveillance are general hospitals and, therefore, are more likely to be representative of the general population than specialty or tertiary care referral hospitals. The option for a SARI proxy case definition identified using ICD-10 codes, instead of applying WHO's clinical case definition (hospitalized patient with fever, cough, and onset of disease within the last 10 days), which would be collected through a questionnaire-based surveillance, was made to minimize the burden of the healthcare professionals [6]. We note that physicians who participated in a study conducted in Portugal with the aim to measure seasonal influenza vaccine effectiveness (EVA hospital study) and primary care clinicians from Portuguese sentinel sites perceived data collection as time-consuming, especially during influenza and COVID-19 epidemic peaks [20, 46]. On the other hand, it is important to note that all reporting procedures for each hospital are automated and routinely programmed, therefore, minimizing workload and guaranteeing timeliness. Additionally, the selected sentinel sites have prior experience in surveillance and at least one hospital has a human resource entirely dedicated to this task, thus, ensuring data quality and commitment to the surveillance system. Finally, the methodology we used to estimate the catchment population can be replicated in other potential sentinel surveillance sites. It is also reassuring that the BIMH database used for these estimates accounts for more than 70% of all national hospital admissions. Finally, stressing the importance of sustainability of this surveillance system, estimating the catchment population did not result in extra work for the hospitals.

Linkage of laboratory with clinical data is being tested as, in the long term, our goal is to establish an integrated and automated SARI syndromic surveillance system combined with laboratory outcomes, in sentinel hospitals evenly geographically distributed across Portugal. This step will aid in understanding the relative contribution of respiratory viruses among SARI cases, especially, when multiple respiratory viruses are cocirculating. Following WHO and ECDC guidelines for respiratory virus surveillance, all specimens taken from SARI sentinel surveillance will be tested at the sentinel sites, using multiplex polymerase chain reaction (PCR) assays to simultaneously detect influenza viruses, SARS-CoV-2, and other respiratory viruses that have a major impact on healthcare systems [6]. Additionally, all sentinel specimens positive for influenza viruses or SARS-CoV-2 will be shared for sequencing at the Portuguese Reference Laboratory for Influenza and other Respiratory Virus, for the purpose of monitoring SARS-CoV-2 variant and influenza strain/lineage circulation [6]. To achieve this goal, it is also necessary to make a continuous effort in order to demonstrate the added value of epidemiological surveillance data to stakeholders within hospital sites. This could be achieved by integrating SARI surveillance in existing hospital programs, such as for monitoring antibiotic or antiviral use and resistance, in order to make surveillance data valuable for public health as well as patient care [47, 48]. The groups of diagnosis used to categorize SARI patients in this study were based on signs and symptoms of respiratory infection (reported in Table 1). Given that within each set, signs or symptoms of respiratory illness may differ in their sensitivity and specificity to detect variations in influenza and COVID-19 infection trends, we plan to study specific diagnosis separately for each disease, and then expand or restrict ICD-10 admission codes in each group when needed. Regarding future steps in data validation analysis, the national register of hospital discharge diagnoses with specific ICD-10 codes related to respiratory infections is available with a considerable time lag, which precludes its use for nearly real-time SARI surveillance. Discharge diagnoses are manually attributed by trained medical professionals and, therefore, using this information for retrospective analysis may help to confirm and validate results from the surveillance system based on hospital diagnosis at admission.

## 5. Conclusion

In the 2021/2022 season, the Portuguese SARI sentinel surveillance system pilot was able to early detect the COVID-19 epidemic peak, in January 2022, and the increase of influenza activity, in March 2022. Although cardiovascular manifestations associated with influenza infection are known, more seasons of SARI surveillance are needed, to confirm the potential use of cardiovascular hospitalizations as an indicator of influenza activity. Combining syndromic surveillance with virological inpatient surveillance will aid in understanding the relationship between respiratory virus epidemics and disease severity.

## Figures and Tables

**Table 1:** Description of sentinel sites participating in SARI surveillance in 2021/2022 (wards, number of beds, and capacity might be updated throughout epidemic peak periods).

Hospital	Characteristics	Number of beds per ward
Centro Hospitalar Universitário Lisboa Central	General hospital	Infectiology: 36 beds
		Medicine: 281 beds

Centro Hospitalar Universitário de São João	General hospital	Infectiology: 23 beds
		Infectiology ICU: 10 beds
		Medicine: 201 beds
		Intensive care unit: 60 beds
		Paediatrics: 36 beds + 6 beds paediatric ICU

**Table 2:** List of diagnosis codes used for severe acute respiratory infection cases identification, hospital-based surveillance.

Category	Morbidity	ICD-10
Influenza-like illness	Cough	R05
	Difficulty breathing	R06
	Sore throat	R07.0
	Dysphagia	R13
	Fever	R50.9
	Headache	R51
	Myalgia	M79.1
	Fatigue/malaise	R53.1, R53.81, R53.83

Cardiovascular diagnosis	Acute myocardial infarction or acute coronary syndrome	I20-23, I24-25
	Heart failure	I50, I51

Respiratory diagnosis	Emphysema	J43.9
	Chronic obstructive pulmonary disease	J44.9
	Asthma	J45
	Dyspnoea/respiratory abnormality	R06.0
	Respiratory abnormality	R06.9
	Shortness of breath	R06.02
	Tachypnoea	R06.82
	Other respiratory abnormalities	R06.00, R06.09, R06.3, R06.89

Respiratory infection	Pneumonia and influenza	J09-J18
	Other acute lower respiratory infections	J20-J22
	Viral infection, unspecified	B34.9
	Bacterial infection, unspecified	A49.9
	Bronchitis	J40, 41
	Myocarditis	I40.9
	COVID-19, virus identified	U07.1
	COVID-19, virus not identified	U07.2

ICD-10: International Statistical Classification of Diseases and Related Health Problems, 10^th^ Revision; COVID-19: coronavirus disease.

**Table 3:** Municipalities and proportion of severe acute respiratory infection cases included in the catchment areas of Centro Hospitalar Universitário de São João (*n* = 12,651 SARI cases) and Centro Hospitalar Universitário Lisboa Central (*n* = 12,536 SARI cases), and respective population under surveillance.

Surveillance site	Centro Hospitalar Universitário de São João	Centro Hospitalar Universitário de Lisboa Central
Number of municipalities	17	12
Proportion of SARI cases	88%	82%
Population under surveillance estimate	434,720	469,435

SARI: severe acute respiratory infection.

**Table 4:** Summary of severe acute respiratory infection cases, stratified by age group and diagnosis, North and Lisbon and Tagus Valley regions, 28 September 2021–22 May 2022 (*n* = 3,563).

	Number	%
Age (in years)		
0–4	159	4.5
5–14	88	2.5
15–44	184	5.2
45–64	565	15.9
65–79	1,011	28.4
≥80	1,556	43.7

Diagnosis		
Total SARI	3,563	—
Influenza-like illness	455	12.8
Cardiovascular diagnosis	986	27.7
Respiratory diagnosis	228	6.4
Respiratory infection	2,082	58.4

SARI: severe acute respiratory infection. The sum of SARI cases by diagnosis category does not equal the total SARI cases, as the case definition for SARI syndrome was based on ICD-10 codes and some codes, can fall into more than one diagnosis category.

**Table 5:** Cross-correlation analysis between severe acute respiratory infection cases (*n* = 3,563), (1) COVID-19 cases (*n* = 2,367,770) and (2) influenza cases (*n* = 4,174). North and Lisbon and Tagus Valley regions, 28 September 2021–22 May 2022.

Lag	(1) COVID-19					(2) Influenza				
	SARI	Influenza-like illness	Cardiovascular diagnosis	Respiratory diagnosis	Respiratory infection	SARI	Influenza-like illness	Cardiovascular diagnosis	Respiratory diagnosis	Respiratory infection
−5	0.38	−0.09	0.10	−0.15	0.35	−0.08	−0.24	0.08	0.08	−0.07
−4	0.50	−0.14	0.04	−0.20	0.50	−0.18	−0.07	0.20	0.12	−0.23
−3	0.62	−0.18	0.00	−0.24	0.64	−0.22	−0.01	0.31	0.11	−0.32
−2	0.71	−0.22	0.06	−0.31	0.73	−0.20	0.08	0.43	0.07	−0.35
−1	0.80	−0.25	0.12	−0.35	0.81	−0.19	0.05	0.44	−0.08	−0.33
**0**	**0.78**	**−0.38**	**0.07**	**−0.37**	**0.82**	**−0.20**	**0.06**	**0.37**	**−0.18**	**−0.31**
1	0.59	−0.45	−0.04	−0.35	0.69	−0.22	0.13	0.24	−0.24	−0.28
2	0.35	−0.49	−0.13	−0.26	0.48	−0.28	0.07	0.10	−0.30	−0.28
3	0.11	−0.37	−0.15	−0.09	0.22	−0.31	0.00	0.03	−0.33	−0.27
4	−0.13	−0.20	−0.09	0.02	−0.07	−0.28	−0.02	0.02	−0.32	−0.24
5	−0.29	−0.09	0.03	0.13	−0.29	−0.25	−0.08	−0.02	−0.33	−0.19

SARI: severe acute respiratory infection; COVID-19: coronavirus disease. Values corresponding to Pearson correlations (lag = 0) are highlighted in bold.

## Data Availability

The data used to support the findings of this study have not been made available becausethe current ethical approval does not permit their deposition.
